# Evaluation of the economic burden of leprosy among migrant and resident patients in Guangdong Province, China

**DOI:** 10.1186/s12879-017-2869-8

**Published:** 2017-12-11

**Authors:** Mingzhou Xiong, Ming Li, Daocheng Zheng, Xiaohua Wang, Ting Su, Yongfeng Chen, Bin Yang

**Affiliations:** 1 Leprosy Control Department, Dermatology Hospital of Southern Medical University, Guangzhou, Guangdong Province China; 2 Leprosy Control Department, Guangdong Provincial Center for Skin Disease and STI Control, Guangzhou, Guangdong Province China

**Keywords:** Leprosy, Economic burden, Migrant, Cross-sectional study

## Abstract

**Background:**

A lot of time and money was needed during the diagnosis and treatment process of leprosy, the delayed leprosy would also impair the labor capability of patients as well, and these put a heavy burden for the leprosy patients. The migrant leprosy patient is a special group and need more concern. Our goal was to assess the economic burden of leprosy on migrant and resident patient populations in Guangdong province, China.

**Methods:**

We conducted a population-based cross-sectional survey from February to July of 2016. A self-designed questionnaire was administered to leprosy patients who: (1) had registered in Leprosy Management Information System in China (LEPMIS) by the end of February 2016, (2) had received multiple drug treatment (MDT) drugs at a local leprosy control institution for three consecutive months or had had at least one physical check in the past half year, and (3) were willing to take part in the investigation and give informed written consent. Demographic characteristics, Financial and disease information, and costs before and after leprosy diagnosis were collected and compared using t-test and χ2 test.

**Results:**

A total of 254 participants completed the questionnaires, including 168 males and 86 females. Migrants and residents accounted for 33.9% and 66.1% of patients, respectively. Among migrant patients, the median cost before diagnosis was $131.6 (39.2–450.9), the median yearly cost of leprosy treatment after diagnosis was $300.6 (158.4–868.5), and the median yearly cost of leprosy complications was $69.5 (11–178.4). In comparison, among residents the median yearly costs were $152.4 (30.7–770.9) pre-diagnosis, $309.7 (103.2–1016.7) after diagnosis, and $91.9 (32.6–303.1) for leprosy complications. Base on this, we determined that the median yearly total expense after diagnosis amounted to 15% of migrant and 38% of resident patients’ annual income.

**Conclusion:**

Leprosy places a heavy economic burden on both migrant and resident leprosy patients and governmental policies and programs could substantially alleviate this. Measures to implement more active surveillance and early diagnosis would benefit both populations, while labor protection and medical insurance are urgently needed for migrant patients and easier access to medical services and social aids could substantially decrease the burden of leprosy for resident patients.

## Background

Leprosy, also known as Hansen’s disease (HD), is a chronic infectious disease caused by the bacillus *Mycobacterium leprae* [1] that results in granuloma formation in the nerves, respiratory tract, skin, and eyes [2]. This may lead to an inability to feel pain and the loss of parts of the extremities due to repeated injuries or infections caused by unnoticed wounds, and the inability occurs especially by leprosy reactions and also specific infiltration of Multibacillary leprosy; it can also cause weakness and poor eyesight [3]. For centuries leprosy has been a major public health problem and this remains true even now: in 2014 alone 213,899 new leprosy cases were reported in 121 countries, and 14,110 new leprosy cases with grade 2 disability (G2D) were reported in 115 countries, raising the global G2D rate from 22% in 2005 to 26% in 2014 [4]. The most severe leprosy endemic in China is in the southeastern province of Guangdong: records show that 96,461 people were affected by leprosy between 1949 and 2015 in Guangdong province alone, accounting for about 20% of all leprosy patients in China.

Guangdong also has the largest migrant population in China. According to the sixth national population census in 2010, about 31.28 million migrants were living in Guangdong province, accounting for 30% of the whole population [5–8]. Migrants have also had an enormous impact on the leprosy endemic in Guangdong province, accounting for 25% of all new leprosy cases from 2004 to 2013 and 38.3% in 2015 [9–12], with 88.0% of these patients living in the Pearl River Delta region alone [13]. Most of resident leprosy patients live in rural regions in eastern, western and northern Guangdong [14, 15].

One of the most difficult problems facing this vulnerable population is the severe economic burden leprosy patients may face, and this issue urgently requires attention. Most patients live in poor areas or have low incomes [16], and newly diagnosed leprosy patients are often forced to leave their current job to receive treatment. Others may lose their jobs because of stigma and discrimination and be prevented from earning a living. While anti-leprosy drugs and laboratory tests are provided for free, patients still need to pay transportation and accommodation fees during monthly visits to local leprosy control institutions. These visits impose further indirect economic costs because of the time lost during treatment. As the disease progresses patients may also suffer from severe complications such as erythema nodosum leprosum (ENL), requiring even more expensive treatments [17]. Although the government has worked over the past few decades to decrease the incidence of leprosy and increase treatment accessibility, it has ignored the economic burden patients face and few studies have focused on this issue. This study aims to assess the economic burdens of leprosy and analyze the differences between the experiences of migrant and resident leprosy patients in Guangdong province to provide information for making policies that minimize the burdens of leprosy.

## Methods

### Study design

We conducted a population-based cross-sectional survey of leprosy patients in Guangdong Province, China from February to July 2016.

### Study site and participants

This study was conducted in Guangdong Province where a total of 294 leprosy patients were receiving treatment at the end of 2015. Leprosy patients can choose a leprosy control institution near their home or place of work for monthly treatment with multiple drug therapy (MDT) over the course of 6 months to 2 years, depending on the type of leprosy. MDT drugs and the corresponding laboratory tests are provided for free to all patients by the government.

All leprosy patients in Guangdong province are registered in the Leprosy Management Information System in China (LEPMIS), and we used this registry to identify counties with at least one leprosy patient as survey study sites. Leprosy patients who (1) had registered in LEPMIS by the end of February 2016, (2) had received MDT drugs at a local leprosy control institution for the past three consecutive months or had had at least one physical check in the past 6 months, and (3) were willing to take part in the investigation and give informed written consent, were recruited for this study. Individuals with complications from other severe diseases or those with logopathy or other expression disorders that prevented them from accurately answering survey questions were excluded. Resident leprosy patients were defined as those receiving treatment at leprosy control institutions in their city of origin and migrant leprosy patients were defined as those receiving treatment in cities other than their city of origin. The Questionnaires were administered to patients by municipal and county leprosy control institutions.

### Participant recruitment and questionnaire administration

Patients visiting the leprosy control institution for a physical check or to receive MDT drugs were asked by the investigator whether they were willing to take part in the questionnaire survey. If written informed consent was given patients were asked to complete the questionnaire under the investigator’s supervision in a private room.

### Data collection

As no validated questionnaire can be used to assess economic burden of leprosy patients, we have designed a questionnaire specifically for this study through Delphi Method. The coefficient of concordances (ω) in the first and second counseling round are 0.315, 0.409, respectively, and the results have been published in a Chinese journal [18]. Patients were asked for information about their demographic characteristics, family structure and financial conditions, levels of leprosy, and the money they lost because of their disease.

**Fig. 1:**
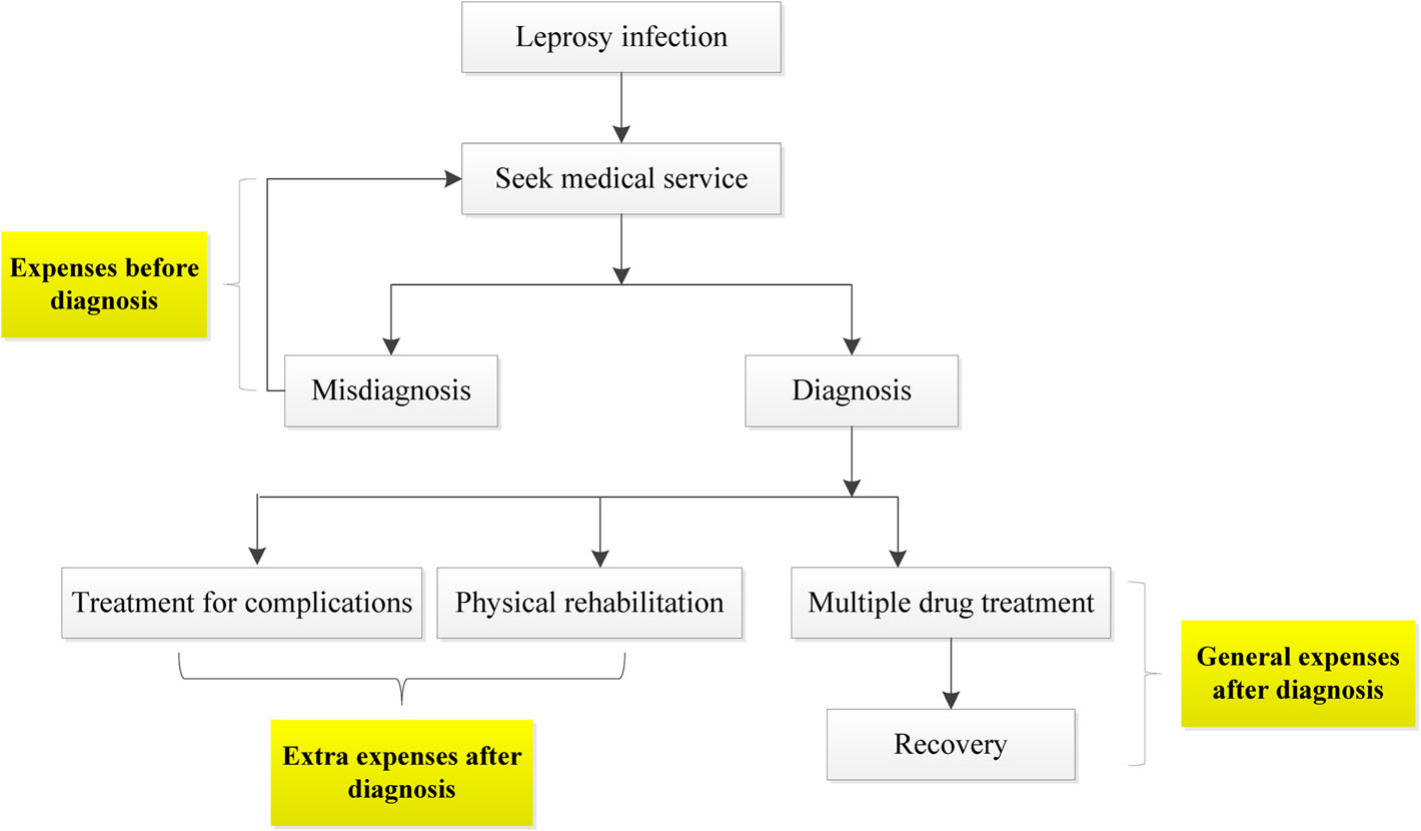
The diagnosis and treatment process for leprosy patients

To obtain detailed information we divided the questions about economic burden into three categories, asking participants to estimate their general expenses before and after diagnosis and the extra expenses incurred after diagnosis. Each category included the costs of medical treatment, transportation to and from treatment centers, housing and food while traveling, and any other relevant fees for each trip. Time loss was measured by the number of days patients spent undergoing in-patient or out-patient treatment or rehabilitation as well as time spent traveling to treatment centers; the economic cost of this lost time was calculated based on the patient’s annual income. Expenses before diagnosis were defined as the time and money lost before the patient could get a correct diagnosis, including the frequency of hospital visits for leprosy symptoms pre-diagnosis. General expenses after diagnosis included the money and time lost for the patient to get the initial leprosy diagnosis and for each subsequent visit to the local leprosy control institution for physical checks and their MDT drugs. Extra expenses after diagnosis were defined as disease complications, such as lepra reactions, as well as the cost of physical and psychological rehabilitation. Because treatment duration varied among the patients we standardized their losses by determining the cost per year. Figure 1 outlines the diagnosis and treatment process for leprosy patients and how the costs were broken into the three categories.

### Measurements and outcomes

As stated above, the expenses before diagnosis measured the loss of time and money for patients seeking medical services for leprosy symptoms before a confirmed leprosy diagnosis. Because patients generally made multiple hospital visits before diagnosis the average cost per visit was multiplied by the number of visits per year and the time cost was then added to this total.

General expenses after diagnosis were similarly determined by calculating the time and money lost by each patient in 1 year to get a leprosy diagnosis, receive their MDT drugs on a regular basis, and have a physical check at their local leprosy control institution. The average cost for each visit was multiplied by the number of treatment-related trips per year and the time cost was added to this total.

Finally, the extra expenses after diagnosis were determined based on travel time and expenses, hospital stays, and medical costs for rehabilitation and treatment of complications; time costs were calculated by the same method used for expenses before diagnosis and general expenses after diagnosis.

The ratio of expenses to income represents the total expenses per year after diagnosis as a portion of patients’ annual income.

### Statistical methods

Epidata 3.0 was used to create the database of patient information and all data were double entered and checked for consistency. Data analysis was performed using Statistic Package for Social Science (SPSS) V.24.0. Proportions, medians, and ratios were used to describe the corresponding indices of this study. T test and analysis of variance (ANOVA) were used to determine statistically significant differences of quantitative indicators and χ2 test was used for qualitative indicators. Results were considered statistically significant if *p* ≤ 0.05.

### Ethics approval and consent to participate

This research has been reviewed and approved by Ethics Review Committee of Guangdong Provincial Center for Skin Disease and STI Control and WHO Western Pacific Region. Each participant was provided written informed consent of the questionnaire investigation.

## Results

### Demographic characteristics

A total of 254 participants completed the questionnaires, including 87 migrants and 167 residents, and their demographics are given in Table 1. About 40 registered patients no concordance in participation because of unwillingness, loss to follow up, and with complications from other severe diseases or those with logopathy or other expression disorders. About 69.0% of migrant and 64.7% of resident patients were male, and 67.8% of migrants and 71.3% of resident patients were married. Most of the migrant patients were between 20 and 40 years old (59.8%), significantly younger than resident patients (*p* < 0.05). Resident patients were less likely to be unemployed (24.1%) than migrant patients (52.7%), even though both groups (52.1% of residents and 41.2% of migrants) had education levels at or below the level of primary school. Resident patients were significantly more likely to be farmers (56.9%) than migrant patients (37.9%, *p* < 0.05) and most non-farmer migrants were factory workers (42.5%). There was no significant difference in annual income for most of the migrant and resident patients: both groups typically earned less than US $7256. (Table 1).

**Table 1:** Demographic characteristic of participants

	Migrants *n* (%)	Residents *n* (%)	Total *n* (%)	χ^2^	*P* Value
Sex					
Male	60 (69.0)	108 (64.7)	168 (66.1)	0.471	0.577
Female	27 (31.0)	59 (35.3)	86 (33.9)		
Age					
< 20	1 (1.1)	9 (5.4)	10 (3.9)	25.769	<0.001
21–40	52 (59.8)	48 (28.7)	100 (39.4)		
41–60	22 (25.3)	54 (32.3)	76 (29.9)		
> 60	11 (12.6)	53 (31.7)	64 (25.2)		
No data	1 (1.1)	3 (1.8)	4 (1.6)		
Education					
Primary school or below	35 (40.2)	87 (52.1)	122 (48.0)	3.783	0.286
Junior high school	31 (35.6)	49 (29.3)	80 (31.5)		
Senior high school	12 (13.8)	20 (12.0)	32 (12.6)		
College and up	8 (9.2)	9 (5.4)	17 (6.7)		
No data	1 (4.9)	2 (1.2)	3 (1.2)		
Marriage					
Single	19 (21.8)	37 (22.2)	56 (22.0)	3.248	0.517
Married	59 (67.8)	119 (71.3)	178 (70.1)		
Divorced	4 (4.6)	2 (1.2)	6 (2.4)		
Widowed	5 (5.7)	8 (4.8)	13 (5.1)		
No data	0 (0.0)	1 (0.6)	1 (0.4)		
Employment					
Full-time	53 (60.9)	48 (28.7)	101 (39.8)	28.418	<0.001
Part-time	4 (4.6)	11 (6.6)	15 (5.9)		
Unemployed	21 (24.1)	88 (52.7)	109 (42.9)		
Retired	2 (2.3)	11 (6.6)	13 (5.1)		
No data	7 (8.0)	9 (5.4)	16 (6.3)		
Profession					
Farmers	33 (37.9)	95 (56.9)	128 (50.4)	25.931	<0.001
Factory workers	37 (42.5)	23 (13.8)	60 (23.6)		
Businessmen	4 (4.6)	12 (7.2)	16 (6.3)		
Public servants	1 (1.1)	6 (3.6)	7 (2.8)		
Others	11 (12.6)	25 (15.0)	36 (14.2)		
No data	1 (1.1)	6 (3.6)	7 (2.8)		
Annual income ($)					
<753 (About 5000 RMB)	15 (17.2)	55 (32.9)	70 (27.6)	7.375	0.061
753–3012 (About 5001–20,000 RMB)	26 (29.9)	44 (26.3)	70 (27.6)		
3012–7530 (About 20,001–50,000 RMB)	23 (26.4)	38 (22.8)	61 (24.0)		
> 7530 (About 50,000 RMB)	10 (11.5)	11 (6.6)	21 (8.3)		
No data	13 (14.9)	19 (11.4)	32 (12.6)		

### Financial and disease information

While 91.6% of resident patients had medical insurance, significantly fewer migrant patients, only 72.4%, (*p* < 0.05) were insured. More than 20% of migrant (21.8%) and resident (26.9%) patients were in debt and 23.0% of migrant patients and 15.0% of resident patients had received financial aid from others, though this difference was not statistically significant. About 29(33.3%) migrant and 69(41.3%) resident patients reported income decrease, while 13(14.9%) migrant and 25(15.0%) resident patients have lost their job after diagnosed with leprosy.

The majority of patients in both groups (47.1% of migrants and 40.1% of residents) had been diagnosed with LL leprosy within 2 years of the symptoms’ appearance (48.3% of migrants, 56.9% of residents). However, more than 6% of patients in each group (6.9% of migrants, 6.6% of residents) were not correctly diagnosed for 10 years (*p* = 0.667). There were no significant differences between migrant and resident populations in the percentage with grade 2 disability (G2D; 13.8% vs. 18.6%), neuritis (28.7% vs 25.1%), or lepra reaction (32.1% vs 22.8%), but migrant patients were significantly less likely to have grade 1 disability (G1D; 8% vs. 16.8%, *p* = 0.043). (Table 2).

**Table 2:** Financial and disease information for migrant and resident leprosy patients

	Migrants *n* (%)	Residents *n* (%)	Total *n* (%)	χ^2^	*P* Value
Medical insurance					
Yes	63 (72.4)	153 (91.6)	216 (85.0)	15.265	<0.001
No	22 (25.3)	13 (7.8)	35 (13.8)		
No data	2 (2.3)	1 (0.6)	3 (1.2)		
Debt					
Yes	19 (21.8)	45 (26.9)	64 (25.2)	0.708	0.400
No	67 (77.0)	122 (73.1)	189 (74.4)		
No data	1 (1.1)	0 (0.0)	1 (0.4)		
Financial aid					
Yes	20 (23.0)	25 (15.0)	45 (17.7)	2.760	0.115
No	60 (69.0)	131 (78.4)	191 (75.2)		
No data	7 (8.0)	11 (6.6)	18 (7.1)		
Income Changing after diagnosed					
Decrease	29(33.3)	69(41.3)	98(38.6)	5.79	0.055
Increase	10(11.5)	7(4.2)	17(6.7)		
Insignificant	40(46.0)	87(52.1)	127(50.0)		
No data	8(9.2)	4(2.4)	12(4.7)		
Job Changing after diagnosed					
Loss job	13(14.9)	25(15.0)	38(15.0)	22.614	<0.001
Go home and be a farmer	1(1.1)	16(9.6)	17(6.7)		
Change job	24(27.6)	20(12.0)	44(17.3)		
Unemployment both before and after diagnosed	9(10.3)	45(26.9)	54(21.3)		
No change	39(44.8)	61(36.5)	100(39.4)		
No data	1(1.1)	0	1(0.4)		
Leprosy type^a^					
TT	4 (4.6)	4 (2.4)	8 (3.1)	13.220	0.033
BT	12 (13.8)	37 (22.2/)	49 (19.3)		
BB	4 (4.6)	13 (7.8)	17 (6.7)		
BL	21 (24.1)	41 (24.6)	62 (24.4)		
LL	41 (47.1)	67 (40.1)	108 (42.5)		
I	3 (3.4)	0 (0.0)	3 (1.2)		
no data	2 (2.3)	5 (3.0)	7 (2.8)		
Delay time					
Two years and below	42 (48.3)	95 (56.9)	137 (53.9)	1.566	0.667
2–5 years	22 (25.3)	33 (19.8)	55 (21.7)		
5–10 years	11 (12.6)	22 (13.2)	33 (13.0)		
10 years and up	6 (6.9)	11 (6.6)	17 (6.7)		
No data	6 (6.9)	6 (3.6)	12 (4. 7)		
G1D^b^					
Yes	7 (8.0)	28 (16.8)	35 (13.8)	3.491	0.043
No	78 (89.7)	138 (82.6)	216 (85.0)		
No data	2 (2.3)	1 (0.6)	3 (1.2)		
G2D					
Yes	12 (13.8)	31 (18.6)	43 (16.9)	0.822	0.479
No	73 (83.9)	135 (80.8)	208 (81.9)		
No data	2 (2.3)	1 (0.6)	3(1.2)		
Neuritis					
Yes	25 (28.7)	42 (25.1)	67 (26.4)	0.485	0.547
No	60 (69.0)	124 (74.3)	184 (72.4)		
No data	2 (2.3)	1 (0.6)	3 (1.2)		
Lepra reaction					
Yes	28 (32.2)	37 (22.8)	66 (26.0)	2.929	0.097
No	57 (65.5)	128 (76.6)	185 (72.8)		
No data	2 (2.3)	1 (0.6)	3 (1.2)		

^a^TT-Tuberculosis leprosy; BT-borderline tuberculoid leprosy; BB-borderline leprosy; BL-borderline lepromatous leprosy; LL-lepromatous leprosy; I-indeterminate leprosy

^b^The leprosy disability grades were in terms of the standard of the sixth leprosy experts committee of WHO (1988)

### Economic burden of leprosy for migrant and resident patients

Both migrant and resident patients visited hospitals an average of three times (IQR 1–6) before receiving a diagnosis, costing an average of 41.3 (IQR 15–105.2) US Dollar for migrants and 45.1 (IQR 18–150.3) US Dollar for residents per visit. Each group spent a median of 3 days for each hospital visit. In total, the median expenses before diagnosis were 131.6 (IQR 39.2–450.9) US Dollar for migrants and152.4 (IQR 30.7–770.9) US Dollar for residents.

The median cost for the diagnosis (include the medical, travel-related, and time loss cost) of leprosy was 75.2 US Dollar for both groups, but with different IQR: $13.5–150.3 for migrants and $25.6–180.4 for residents. For migrant patients, the median time between diagnosis and the start of drug treatment at a local leprosy institution was 20 (IQR 8.5–29) days, similar to the resident median time of 15 (IQR 8–27) days. The median time loss for receiving treatment after diagnosis was also similar: 13.5 (IQR 7–17.5) days for migrants, and 12 (IQR 6–17) days for residents. Interestingly, the average cost of receiving treatment each time was slightly lower for migrant patients (9 US Dollar, IQR 3.8–30.1) than for resident patients (15 US Dollar, 6.8–30.1) but the median general expense per year after diagnosis was similar: 300.6 (IQR 158.4–868.5) US Dollar for migrants and 309.7 (IQR 103.2–1016.7) US Dollar for residents.

After diagnosis, the median extra expenses per year were 69.5 (IQR 11–178.4) US Dollar for migrant and 91.9 (IQR 32.6–303.1) US Dollar for resident patients, resulting in a median yearly total expense of 471.0 (IQR 209.7–1229.2) US Dollar for migrants and 562.9 (IQR 255.7–1295.8) US Dollar for residents after diagnosis.

Although the median ratio of total expenses after diagnosis per year to annual income was lower for migrant (0.15, IQR 0.04–0.50) than for resident (0.38, IQR 0.13–1.45) patients the difference was not statistically significant (*p* > 0.05). (Table 3).

**Table 3:** Economic burden of leprosy for migrant and resident patients

	Migrant (*N* = 87) median (IQR)	Resident (*N* = 167) median (IQR)	Total median (IQR)	t	*P* value
Expenses before diagnosis					
Hospital visits (N)	3 (1–6)	3 (1–6)	3 (1–6)	1.268	0.206
Average cost for each visit ($)	41.3 (15–105.2)	45.1 (18–150.3)	45.1(15–120.2)	0.558	0.577
Time lost for each visit (days)	3 (1–8)	3 (1–10)	3 (1–10)	0.897	0.371
Total expenses before diagnosis per year ($)	131.6 (39.2–450.9)	152.4 (30.7–770.9)	151.7 (35.6–677.4)	1.341	0.147
General expenses after diagnosis					
Cost for diagnosis ($)	75.2 (13.5–150.3)	75.2 (25.6–180.4)	71.2 (15–180.4)	0.458	0.648
Visits to receive drugs (n)	20 (8.5–29)	15 (8–27)	17 (8–27.5)	0.253	0.801
Average cost for each visit ($)	9 (3.8–30.1)	15 (6.8–30.1)	13.5 (4.5–30.1)	0.357	0.721
Time lost for each visit (days)	13.5 (7–17.5)	12 (6–17)	12.5 (6.5–17.5)	0.273	0.785
Total ordinary expenses after diagnosis per year ($)	300.6 (158.4–868.5)	309.7 (103.2–1016.7)	356.2 (180.4–876.6)	0.119	0.905
Extra expenses after diagnosis					
Extra expenses per year ($)	69.5 (11–178.4)	91.9 (32.6–303.1)	87.5 (26.9–266.7)	1.160	0.247
Total expense after diagnosis per year ($)	471.0 (209.7–1229.2)	562.9 (255.7–1295.8)	515.4 (238.3–1275.9)	1.054	0.293
Ratio of expense to income	0.15 (0.04–0.50)	0.38 (0.13–1.45)	0.31 (0.09–1.02)	0.702	0.483

## Discussion

As the endemic of leprosy has been brought under control, dropping to fewer than 0.1 cases per 100,000 people in Guangdong province in 2011, both government and society at large have turned their attention away from this issue. However, discrimination against leprosy remains ubiquitous. Leprosy patients encounter social stigma that drives them to conceal their disease from neighbors and even family members, and are faced with the burdens of treatment costs and lost wages while still needing to feed their families.

Compared to residents, migrants are typically younger and driven to urban areas in search of manufacturing jobs [19, 20]. We found that most migrant patients were 20–40 years old and employed full-time in factories, while resident patients were over 40 years old and were typically farmers or were unemployed. In contrast to previous research reporting that migrants were more likely to earn a lower salary than residents in both urban and rural areas [21], migrant patients in our study reported a higher average annual income than resident patients, though the difference was not statistically significant. One explanation for this may be that patients no longer able to work were more likely to return to their hometown for treatment. Our data also indicates a significantly lower annual income for migrant patients in Guangdong than has been reported for the whole migrant population in China [22]; we suspect the low education level and lost work time caused by leprosy may lead to this difference. Because migrants are thus more likely to earn marginal incomes, increased attention should be paid to migrants who do not have medical insurance, as these families are at an increased risk of returning to poverty as a result of serious illness.

During the early course of disease, leprosy patients often face misdiagnosis because the various incipient symptoms can be non-specific [23, 24] and this increases both the disease and economic burden before patients get a correct diagnosis. After diagnosis, monthly visits to local leprosy institutes for drugs consumes even more time and money. Both before and after diagnosis we observed a relatively higher cost for resident than for migrant patients, although the difference was not statistically significant. This may appear counter-intuitive at first, since the same amount of time lost for treatment equates to a higher cost for migrant patients with a full-time job and a relatively higher salary. Nevertheless, expenses before and after diagnosis accounted for 38% of average yearly income for residents and only 15% for migrants, most likely because migrants working in factories receive higher average incomes. Another factor may be that resident patients on average were significantly older with more severe leprosy outcomes, including development of G1D, resulting in higher treatment costs. As a result, migrants face a relatively lower economic burden on average.

Now that the leprosy endemic is better controlled there needs to be a shift of focus towards improving patients’ quality of life, and decreasing the economic burden of the disease is a crucial step in this process. Diagnosis of leprosy during its early stages is highly effective in lowering pre-diagnosis costs as well as preventing progressive disability and preserving a patient’s ability to work. A passive surveillance system has been used for leprosy control in recent years, but instituting active surveillance would likely be more effective as disease endemic decreases [25–28]. Zhejiang province recently adopted a system that closely monitored possible leprosy symptoms, enabling diagnosis at early disease stages that resulted in improved control of the endemic as well as a better cost-benefit ratio [29]. This system could easily be introduced to Guangdong and other endemic areas as well.

The needs of migrant and resident leprosy patients can vary and may require different approaches. The majority of migrant patients have full-time jobs working long hours in unprotected environments that can accelerate the development of skin lesions and peripheral nerve damage, leading to skin ulcers or physical disabilities. Physicians treating these patients should take this into account and give patients self-care advice based on their occupation to help them reduce their medical costs. An additional burden for migrant workers is their limited access to medical insurance: the localized management policy in China only allows people to participate in rural cooperative medical systems or urban health insurance programs in their registered residences, preventing migrants from obtaining insurance in the cities in which they work but are not residents [30]. In this respect our findings are in line with the rate of insurance among the whole migrant population in China in 2012, 72.4% vs. 69.4% [31], and are lower than the proportion in 2015, which was 89.3% [32]. Current measures are being taken by the Chinese government to promote integration of medical insurance systems across the whole country, and if successful, this may decrease the economic burden on migrant leprosy patients in the future. However, for resident leprosy patients, especially those living in rural villages, the more pressing issue is to improve medical facilities and services. For elderly and disabled patients in particular new approaches to treatment delivery like physician follow-up by phone, sending MDT drugs by express delivery, and regular healthcare worker visits to patients’ houses for disease checks would substantially decrease the economic burden and increase quality of life. While, the above-mentioned suggestions were put forward based on the research results and experiences of the research team, and the feasibility and effectiveness should be proved by further researches.

## Study limitations

Despite our efforts to recruit all the leprosy patients in Guangdong province for this study only 254 participants completed the survey, and the restricted sample size may mean our survey lacked the power to identify statistically significant differences between migrant and resident patients. Recall bias may also have been an issue, even though the investigators administering the questionnaire were doctors in direct supervision of these patients who were very familiar with the participants’ diagnosis and treatment history and could help them recall information more accurately. While our goal was to obtain as much economic data as possible, we also wanted to minimize the burden of memory recall on participants and avoid asking for excessive detail. As a result we were only able to determine the total economic burden and the direct and indirect economic burden could not be calculated. Finally, it was necessary to standardize the expense burden for 1 year based on expenses in previous months in order to include patients who had been treated for less than 1 year.

## Conclusion

Both migrant and resident leprosy patients face heavy economic burdens and the government should take measures to diagnose leprosy patients in the early stages of disease to minimize this burden. Moreover, labor protection and medical insurance are urgently needed for migrant patients and easier access to medical services and social aids should be provided for resident patients; both measures could substantially reduce the economic burden on these populations.

## Abbreviations

ANOVA: Analysis of variance
ENL: Erythema nodosum leprosum
G2D: Grade 2 disability
HD: Hansen’s disease
LEPMIS: Leprosy Management Information System in China
MDT: Multiple drug treatment
SPSS: Statistic Package for Social Science
